# Unified Depth-Guided Feature Fusion and Reranking for Hierarchical Place Recognition

**DOI:** 10.3390/s25134056

**Published:** 2025-06-29

**Authors:** Kunmo Li, Yongsheng Ou, Jian Ning, Fanchang Kong, Haiyang Cai, Haoyang Li

**Affiliations:** 1 School of Control Science and Engineering, Dalian University of Technology, Dalian 116024, China; likunmo@mail.dlut.edu.cn (K.L.); kongfanchang@mail.dlut.edu.cn (F.K.); haiyangcai@mail.dlut.edu.cn (H.C.); lihaoyang0517dlut@mail.dlut.edu.cn (H.L.); 2 School of Computer Science, Wuhan University, Wuhan 430072, China; ningjian@whu.edu.cn

**Keywords:** visual place recognition, depth information, multimodal feature fusion, reranking

## Abstract

Visual Place Recognition (VPR) constitutes a pivotal task in the domains of computer vision and robotics. Prevailing VPR methods predominantly employ RGB-based features for query image retrieval and correspondence establishment. Nevertheless, such unimodal visual representations exhibit inherent susceptibility to environmental variations, inevitably degrading method precision. To address this problem, we propose a robust VPR framework integrating RGB and depth modalities. The architecture employs a coarse-to-fine paradigm, where global retrieval of top-N candidate images is performed using fused multimodal features, followed by a geometric verification of these candidates leveraging depth information. A Discrete Wavelet Transform Fusion (DWTF) module is proposed to generate robust multimodal global descriptors by effectively combining RGB and depth data using discrete wavelet transform. Furthermore, we introduce a Spiking Neuron Graph Matching (SNGM) module, which extracts geometric structure and spatial distance from depth data and employs graph matching for accurate depth feature correspondence. Extensive experiments on several VPR benchmarks demonstrate that our method achieves state-of-the-art performance while maintaining the best accuracy–efficiency trade-off.

## 1. Introduction

As a fundamental capability for autonomous driving systems, Visual Place Recognition (VPR) has become an increasingly important research focus in recent years [1,2]. In robotics, this task is termed loop closure detection, while in computer vision, it is typically formulated as an image retrieval problem [3,4]. The standard VPR task resolution pipeline consists of three main steps: (1) generating distinctive image descriptors for both reference and query image sets, (2) computing similarity metrics between query and reference descriptors, and (3) producing an ordered retrieval list for each query image based on similarity scores. VPR faces many real-world challenges, including appearance variations, viewpoint shifts, perceptual aliasing, and dynamic object interference [5,6].

Most of the current VPR methods focus on the application of RGB information, addressing environmental variations through the construction of robust place descriptors. Some works [7,8,9,10,11] achieve query image retrieval by aggregating local features into compact global descriptors. Some studies [12,13,14] enhance the robustness of global features by improving the feature aggregation layer. However, global feature-based VPR methods demonstrate limited performance when handling scenarios with significant domain shifts. The reranking methods integrate global and local features, utilizing spatial verification and other techniques to optimize global retrieval results, thereby improving VPR accuracy [15,16]. However, conventional reranking approaches typically rely on computationally intensive RANSAC [17] procedures. Several studies [18,19,20] have improved the computational efficiency while achieving high accuracy through the improvement of the local feature matching mechanism. However, these global-feature- and reranking-based approaches solely utilize the single RGB modal information, exhibiting limited accuracy and robustness when handling complex environmental variations.

Scene depth information provides geometrically stable structural representations that have been extensively applied in SLAM systems [21,22,23]. Deep learning-based monocular depth estimation has emerged as a prominent research focus in computer vision, owing to its cost-effectiveness and operational flexibility. Some approaches [24,25] learn scene geometric representations from depth images to construct robust retrieval descriptors. These methods effectively address visual differences in outdoor visual localization tasks. However, the above methods typically employ simple concatenation of RGB and depth features, failing to capture the hierarchical relationships between spatial structures (e.g., building contours) and semantic contexts (e.g., road textures). This architectural limitation results in performance degradation under significant viewpoint variations. Refs. [26,27] utilize geometric constraints from depth data to enhance feature geometric awareness, thereby improving VPR robustness against appearance and viewpoint variations. However, the aforementioned methods primarily employ depth information for feature enhancement or filtering, while neglecting its potential for explicit geometric consistency verification (e.g., RANSAC-based validation). This limitation results in insufficient geometric constraints during the matching phase, thereby compromising the robustness of place recognition. Inspired by the above works, we propose a multimodal network framework that effectively integrates semantic information from RGB images with geometric features from depth images. Meanwhile, we employ a two-stage retrieval pipeline and utilize the geometric verification of depth information as the reranking stage to further enhance the robustness and accuracy of VPR under challenging environmental variations. Notably, our framework maintains strong relevance to sensor technologies in two key aspects: (1) the method processes RGB images (from monocular cameras) paired with depth information, which can be either estimated algorithmically or obtained from depth sensors like LiDAR, and (2) the design specifically targets robotic systems where visual sensors serve as primary perception tools, with depth enhancing geometric robustness against environmental variations.

In summary, our key contributions are as follows:

(1) We propose a robust two-stage VPR framework that effectively integrates RGB and depth information, employing multimodal feature descriptors for global retrieval and utilizing depth-based geometric verification during reranking.

(2) We propose a Discrete Wavelet Transform Fusion (DWTF) module, which incorporates discrete wavelet transform to capture high-frequency details and low-frequency structural information from depth features, thereby generating robust multimodal feature descriptors.

(3) We propose a Spiking Neuron Graph Matching (SNGM) module, which utilizes the attention generated by Spiking Neural Networks to generate discriminative features while incorporating graph matching to achieve precise matching.

(4) Extensive experiments conducted on several VPR benchmarks demonstrate that our method outperforms state-of-the-art methods while achieving the best trade-off between precision and efficiency.

## 2. Related Works

### 2.1. Visual Place Recognition

The single-stage VPR methods typically employ global descriptors to perform the global retrieval. Methods based on VLAD have been proposed successively, such as NetVLAD [7], CRN [8], SFRS [9], APPSVR [10], SRALNet [11], and VLAD-Buff [28]. These approaches aggregate local features into global features by means of feature aggregation to address VPR. Studies on VPR datasets like CosPlace [29], Conv-AP [13], GPM [30], MixVPR [12], EigenPlaces [31], and GCL [32] improve place recognition accuracy by leveraging newly proposed large-scale datasets or refining label annotations in existing ones. Moreover, several studies improve the generalization capability through the optimization of global descriptors. For instance, SALAD [33] enhances descriptor quality by introducing a “dustbin” clustering mechanism. CricaVPR [34] improves model robustness by strengthening the perception ability of inter-image correlations. BoQ [14] generates compact global descriptors through the dynamic aggregation of local features and multi-block cascade architecture. However, the aforementioned single-stage approach exhibits limited robustness to environmental variations and is susceptible to challenges such as perceptual aliasing in complex scenarios.

In recent years, two-stage VPR, namely hierarchical visual place recognition, has emerged as an important research direction. DELG [35] employs a unified architecture to jointly learn global and local image features, achieving state-of-the-art performance in image retrieval tasks. Similarly, works that use global features for global retrieval and local features for subsequent reranking have emerged successively, such as Patch-NetVLAD [15], GeoWarp [36], TransVPR [16], and Pair-VPR [37]. To improve the efficiency of two-stage VPR, recent methods like R2Former [18], SelaVPR [19], and DHE-VPR [20] have optimized the reranking pipeline by replacing traditional RANSAC verification with more efficient alternatives, significantly reducing time consumption while enhancing the accuracy. However, the above single-stage and two-stage methods only rely on the single RGB modality, lacking robustness to challenging environmental variations. Depth images are employed in VPR tasks due to their visual characteristics and geometric information. Ref. [26] has developed a VPR system that integrates depth information with temporal sequence data, effectively addressing the challenges posed by simultaneous changes in environmental appearance and observation viewpoint. Refs. [24,25] use depth information as an auxiliary mode to enhance the robustness of image descriptors and reduce the localization errors caused by challenges such as illumination and seasonal variations. However, the previously mentioned methods neglect the high-frequency and low-frequency information of the image. Our approach employs discrete wavelet transform (DWT) to facilitate multimodal feature fusion, effectively capturing feature distribution, geometric details, and other information from RGB and depth modalities, thereby significantly enhancing the robustness of the multimodal descriptors.

### 2.2. Graph Matching

With the advancement of Graph Neural Networks [38], graph matching offers insights for feature matching by learning more robust features and establishing correspondence relationships between points and edges. Some studies [39,40] utilize the idea of supervised training to address the allocation problem in graph matching by establishing end-to-end mapping relationships. The model based on graph theory addresses the second-order correlation issue between edges within the graph, namely the quadratic allocation problem [41], by means of exploring the optimization method [42,43]. Ref. [44] designed the GST algorithm, which effectively reduced the time consumption for solving the quadratic allocation problem. Unlike SuperGlue [45], which employs Sinkhorn [46] for corner matching, this method achieves rapid convergence by utilizing a differentiable graph matching network. Ref. [47] developed a momentum distillation strategy, which mitigates the adverse impact of dual error associations of nodes and edges on the graph matching accuracy. Ref. [48] utilized the attention mechanism to dynamically predict the number of internal points to solve the partial graph matching problem with outliers. Ref. [49] effectively reduced the impact of landmark variations on VPR accuracy in dynamic environments by leveraging graph matching. Our method effectively integrates the advantages of Spiking Neural Network and graph matching. By focusing on and matching the discriminative features within depth images, it improves the reranking accuracy of VPR while reducing time consumption.

## 3. Method

**Figure 1:**
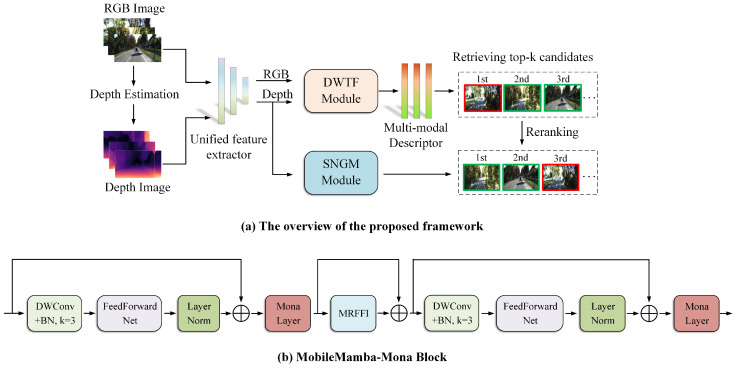
The overall framework of the proposed method and the MobileMamba-Mona Block.

Our method follows the idea based on two-stage VPR and designs two branches: global retrieval and reranking. Specifically, as illustrated in Figure 1, the RGB image is processed via a lightweight self-supervised monocular depth estimation method to obtain the corresponding depth image. Subsequently, the RGB image and the depth image are processed through a unified feature extractor to obtain the corresponding global features. The global features from the two branches are fused via the DWTF module to obtain multimodal features, subsequently yielding preliminary global retrieval results. The features from the deep branch are refined via the SNGM module, and the global retrieval list is reranked to obtain the final retrieval result.

### 3.1. Acquisition of Depth Images

Our method is compatible with both sensor-derived and algorithm-estimated depth data. For systems equipped with depth sensors (e.g., LiDAR or RGB-D cameras), raw depth measurements can be directly fed into the pipeline. When only monocular cameras are available, we employ monocular depth estimation to estimate depth from RGB sequences. For the second situation, the common method for obtaining corresponding monocular depth estimation images from RGB images is Monodepth2 [50]. However, the number of parameters in its model is relatively large, and it fails to capture rich global–local features. Therefore, our framework utilizes the lightweight Lite-Mono method [51] to obtain the depth image corresponding to the RGB image. Lite-Mono effectively integrates the advantages of a CNN and a Transformer. It employs the local–global feature interaction module to encode global information into multi-scale local features, performing well in terms of both the number of parameters and real-time performance of the model. In the experiments of this paper, we adopt the Lite-Mono-tiny model provided in the original paper. This model was trained on the KITTI dataset [52] and can obtain high-quality depth images with merely 2.2 M parameters.

### 3.2. Acquisition of Global Features

After obtaining the depth image, both the RGB image and the depth image are processed through a unified feature extractor to obtain their respective global features. Inspired by MobileMamba [53], we utilize its lightweight and multi-receptive field backbone network for feature extraction. Drawing on the working principle of MobileMamba, we adopt its three-stage network design concept in the coarse-grained design of the feature extractor to achieve higher inference speed and quality. In terms of the fine-grained design, the RGB images and their corresponding depth images first pass through the 16 × 16 downsampled PatchEmbed, followed by the MobileMamba Block and the downsampling module in stages 1 and 2. Different from the work of MobileMamba, to enable the designed unified feature extractor to better capture the feature distribution of the image and enhance training efficiency, we improve the Block of the third stage of MobileMamba and obtain the MobileMamba-Mona Block. The specific structure is illustrated in Figure 1b.

The Block in the original MobileMamba is composed of the Multi-Receptive Field Feature Interaction (MRFFI) module and symmetrically distributed local information perception and FeedForwardNet (FFN). Inspired by Mona [54], by embedding Mona Layer and Layer Norm into the MobileMamba Block, we obtain a new symmetrical structure design. The MobileMamba-Mona Block can achieve efficient fine-tuning while enhancing the processing capacity for visual information. For the Mona Layer, suppose the input to the scaled LayerNorm is $X_{1}$ and the output is $X_{2}$. The relationship between its input and output can be expressed as

(1) $$X_{2}=s_{1}\cdot Layer\;Norm\left(X_{1}\right)+s_{2}\cdot X_{1}$$

where $Layer\;Norm\left(\cdot \right)$ represents the operation of LayerNorm. $s_{1}$ and $s_{2}$ are learnable weight parameters.

Furthermore, if the final output of the Mona Layer is $X_{3}$, then the input–output relationship of the Mona Layer can be expressed by the following formula:

(2) $$X_{3}=X_{1}+u^{i}\sigma \left(f_{pw}\left(f_{dw}\left(d^{i}\left(X_{2}\right)\right)\right)\right),i=1,2$$

where $u^{i}$ and $d^{i}$ represent the upper and lower projection in the Mona Layer, respectively. *i* represents the i-th adapter and σ represents GeLU activation. $f_{pw}$ and $f_{dw}$, respectively, denote the operations related to point-wise convolution and depth-wise convolution in the Mona Layer.

### 3.3. Discrete Wavelet Transform Fusion (DWTF) Module

**Figure 2:**
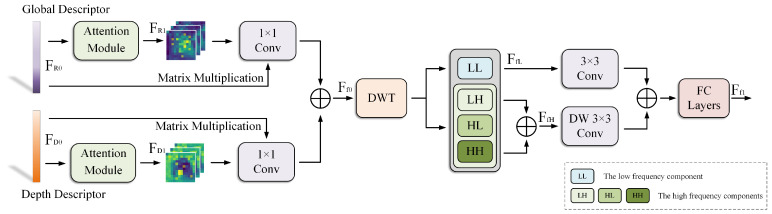
The structure of the Discrete Wavelet Transform Fusion (DWTF) module.

This section introduces the Discrete Wavelet Transform Fusion (DWTF) module for RGB features and depth features, as illustrated in Figure 2. Unlike traditional downsampling, DWT avoids aliasing artifacts and retains structural integrity, as demonstrated in [55,56]. The Discrete Wavelet Transform (DWT) is well suited for feature fusion due to its multi-scale representation, which preserves both global structures and fine details, and its energy compaction property, which emphasizes salient information while reducing redundancy [57]. Inspired by AdaFusion [58] and FFAGRNet [59] on feature fusion, we design the DWTF module to obtain more robust multimodal features. Unlike AdaFusion and FFAGRNet, we focus on fusing RGB features with depth features. The RGB feature $F_{R0}$ and depth feature $F_{D0}$, after passing through the unified feature extractor, are respectively processed by the Attention Module to obtain the corresponding attention features $F_{R1}$ and $F_{D1}$. The Attention Module consists of three distinct depth-wise separable convolutions, namely 3×3, 5×5, and 7×7. The features from these three branches are output after concatenation and ReLU activation. $F_{R1}$ and $F_{D1}$, respectively, undergo 1×1 convolution and then undergo matrix multiplication with the original features $F_{R0}$ and $F_{D0}$. The features obtained from the aforementioned operations are concatenated to obtain the initially fused features $F_{f_{0}}$. The above process can be expressed as

(3) $$F_{f_{0}}=\left(Conv_{1\times 1}\left(F_{R1}\right)⊗F_{R0}\right)⊕\left(Conv_{1\times 1}\left(F_{D1}\right)⊗F_{D0}\right)$$

where $Conv_{1\times 1}\left(\cdot \right)$ denotes 1×1 convolution.

To enable the fused multimodal features to simultaneously capture the high-frequency details and low-frequency structures of the image, we draw inspiration from the Haar wavelet [60]. After $F_{f_{0}}$ is optimized by DWT, the feature of the low-frequency part is $F_{f_{L}}$, and the feature of the high-frequency part after concatenation is $F_{f_{H}}$. $F_{f_{L}}$ and $F_{f_{H}}$ are, respectively, processed with 3×3 Conv and 3×3 Depth-Wise Convolution (DWConv), and then the final multimodal feature $F_{f_{1}}$ is obtained through FC Layers. The aforementioned process can be expressed as

(4) $$F_{f_{1}}=MLP\left(\left(Conv_{3\times 3}\left(F_{f_{L}}\right)\right)⊕\left(DWConv_{3\times 3}\left(F_{f_{H}}\right)\right)\right)$$

where $Conv_{3\times 3}\left(\cdot \right)$ and $DWConv_{3\times 3}\left(\cdot \right)$ denote 3×3 Conv and 3×3 DWConv, and MLP(·) denotes the operation of FC Layers.

### 3.4. Spiking Neuron Graph Matching (SNGM) Module

**Figure 3:**
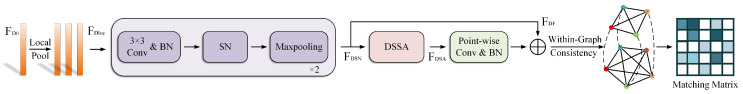
The structure of the Spiking Neuron Graph Matching (SNGM) module.

The architecture of the Spiking Neuron Graph Matching (SNGM) module is illustrated in Figure 3. The original deep feature $F_{D_{0}}$ is obtained after local pooling with GeM. To perform efficient encoding on $F_{Dloc}$ and feature filtering on the noise in $F_{Dloc}$, we introduce Spiking Neural Networks (SNNs). SNNs are employed in the SNGM module due to their event-driven processing and energy-efficient attention mechanisms, which naturally fit depth feature filtering. Unlike traditional CNNs that process all pixels uniformly, SNNs selectively activate only relevant depth edges (e.g., building contours), reducing computational overhead while preserving geometric structures. After $F_{Dloc}$ undergoes a two-layer convolution and pooling structure, $F_{DSN}$ is obtained, where BN represents batch normalization layer and SN represents Spiking Neuron. To focus on the discriminative features in local feature $F_{DSN}$, inspired by SpikingResformer [61], we introduce the Dual Spike Self-Attention (DSSA) module. The DSSA module generates the corresponding attention by incorporating adaptive scaling factors and staged convolutions. Meanwhile, it can significantly reduce computational consumption. After $F_{DSN}$ is optimized by the DSSA module, $F_{DSA}$ is obtained. $F_{DSA}$ is concatenated with $F_{DSN}$ after passing through the point-wise convolution and BN module to obtain $F_{DF}$. The aforementioned process can be expressed as

(5) $$F_{DF}=F_{DSN}⊕\left(BN\left(Conv_{pw}\left(F_{DSA}\right)\right)\right)$$

where $BN(Conv_{pw}\left(\cdot \right))$ denotes the operation of point-wise convolution and BN.

Thanks to the fact that graph matching can explicitly model the relationship between scene features, we introduce the concept of graph matching, thereby effectively utilizing information such as geometric structure and spatial distance contained in the scene within the depth map. Meanwhile, in the graph matching problem, we notice that poor image discriminability or differences in viewpoints and appearances between images lead to matching noise, which interferes with the effective associations between corresponding nodes and edges. Inspired by [47], we introduce the within-graph consistency to constrain the matching of depth images and subsequently generate the matching matrix to achieve precise matching. Specifically, given the key points $X_{A}$ and $X_{B}$ in images A and B, respectively, the corresponding intra-graph edge consistency loss can be expressed as

(6) $$L=\left|\right|X_{A}X_{A}^{T}-X_{B}X_{B}^{T}{\left|\right|}_{F}^{2}$$

where $L=\left|\right|\cdot {\left|\right|}_{F}$ denotes the Frobenius norm and $X_{A},X_{B}\in R^{n\times d}$; *n* denotes the number of key points; and *d* denotes the dimension of the key point features.

## 4. Experiment

In this section, we present a series of comparative experiments conducted with state-of-the-art methods to validate the effectiveness of the proposed method. We present the experimental datasets, compared methods, evaluation metrics, and implementation details, respectively. Furthermore, we present the comparative experiments performed on both VPR benchmark and loop closure detection datasets, followed by comprehensive ablation studies.

### 4.1. Experimental Preparation

#### 4.1.1. Datasets

We adopted several benchmark datasets commonly utilized in VPR to evaluate the performance of various methods, including Mapillary Street-Level Sequences [62], Pitts30k [7], and Tokyo 24/7 [63].

The Mapillary Street-Level Sequences (MSLS) dataset is a large-scale VPR dataset, comprising 1.6 million images of urban and suburban scenes from 30 cities spanning six continents. It encompasses various types of environmental variations, including weather, seasons, day and night conditions, viewpoints, dynamic objects, and changes in building structures. We tested two parts of it: the validation set and the test set.

The Pittsburgh dataset [64] comprises 250k images collected via Google Street View. The reference images and query images were captured at various times of the day and span several years. For each place, a total of 24 images were generated from different angles, comprising two elevation angles and twelve yaw angle directions, resulting in huge variations in viewpoint and appearance. Pitts30k is a subset of the Pittsburgh dataset, comprising 10k reference images and 6816 query images.

The Tokyo 24/7 dataset is a challenging VPR dataset from the center of Tokyo, created via Google Street View. The query database consists of photos captured by smartphones, totaling 315 query images, comprising daytime, dusk, and nighttime images from 105 places.

All benchmark datasets (MSLS, Pitts30k, and Tokyo 24/7) contain sensor-collected data, including RGB images from calibrated cameras and precise GPS ground truth. These sensor-originated data ensure that our evaluation reflects real-world operating conditions. The detailed information of the datasets is shown in Table 1.

#### 4.1.2. Compared Methods

We evaluated the proposed method’s performance through comparisons with several state-of-the-art VPR methods. The comparison methods include single-stage methods using global retrieval, such as NetVLAD, SFRS, GCL, and SelaVPR (global), as well as two-stage methods with reranking, such as SP-SuperGlue [45,65], Patch-NetVLAD, TransVPR, R2Former, and SelaVPR.

#### 4.1.3. Evaluation Metrics

We adopted the same evaluation metric as Patch-NetVLAD, namely the Recall@N. The threshold setting rules are as follows: translation error of 25 m for Pitts30k and Tokyo 24/7; translation error of 25 m and orientation error of 40° for MSLS.

#### 4.1.4. Implementation Details

All images were resized to a resolution of 640×480 prior to training and testing. Our method employed the AdamW optimizer [66] for network training. For the global retrieval stage, the momentum was set to 0.5, the weight decay was set to 0.001, and the loss function employed was the triplet loss [67]. The initial number of training epochs was set to 100, the initial learning rate was set to 0.001, and the learning rate was multiplied by 1/3 every 5 epochs. For the reranking stage, the initial learning rate was set to 0.0001, with the learning rate multiplied by 0.5 every 5 epochs. The initial number of training epochs was set to 30. Training was terminated when the performance did not improve within 3 epochs. We trained and tested our method using the PyTorch framework on a single NVIDIA GeForce RTX 3090 GPU with 24 GB of memory. Following Patch-NetVLAD, we trained on Pitts30k and tested in urban scenarios (Pitts30k and Tokyo 24/7 datasets), and trained on MSLS to test in the remaining scenarios.

### 4.2. Comparisons with State-of-the-Art Methods

#### 4.2.1. VPR Benchmarks

The comparison results of our method with state-of-the-art single-stage and two-stage methods are shown in Table 2, where our method is denoted as Ours (global) when using only global retrieval. For the fairness of comparison, all two-stage methods rerank the top-100 candidate images generated by their respective single-stage counterparts to produce the final results. From the results in the table, it is evident that our method achieves the best results across all VPR benchmarks.

When using only global features for retrieval, ours achieves the highest R@1 across all four datasets. Our method achieves suboptimal performance in terms of R@5 and R@10 on the Pitts30k dataset, and for the Tokyo 24/7 dataset, ours achieves suboptimal performance on R@10. Compared with NetVLAD, our single-stage method (Ours) (global) achieves an average absolute improvement of approximately 27% on R@1 across the four benchmark datasets. When compared to SelaVPR, it yields an approximately 4% absolute gain on R@1 on average. This demonstrates the excellent robustness of our multimodal global descriptor, effectively addressing the challenges of various scenarios.

When reranking is used, our method achieves the best R@1/R@5/R@10. Compared with Patch-NetVLAD and SelaVPR, our approach achieves an absolute improvement of 16% and 4% on R@1, respectively. Benefiting from robust multimodal global features and the excellent reranking strategy, our method exhibits excellent generalization ability and achieves absolute performance advantages on VPR benchmark datasets.

#### 4.2.2. Latency and Memory

Time consumption and memory usage are crucial metrics for evaluating VPR methods. As shown in Table 3, compared with several state-of-the-art two-stage methods, we select the MSLS val dataset to test the feature extraction time, matching time consumption, and memory usage, respectively. From the data in the table, it is evident that our method achieves the best performance in terms of feature extraction and matching latency, outperforming the other two-stage methods in terms of time efficiency. Specifically, the feature extraction time of our method on a single query is 68.9 times faster than that of Patch-NetVLAD and 1.4 times faster than that of $R^{2}\mathrm{Former}$. In terms of matching time consumption for a single query, that of SP-SuperGlue is 84.4 times our method’s, and that of SelaVPR is 1.7 times our method’s. In terms of memory usage, the memory consumption of our method per image is 0.29 MB, which is of the same order of magnitude as that of $R^{2}\mathrm{Former}$ and SelaVPR. Specifically, the memory consumption of Patch-NetVLAD is 152.2 times that of our method, and $R^{2}\mathrm{Former}$ is more lightweight than our method, occupying only 0.27 MB.

**Figure 4:**
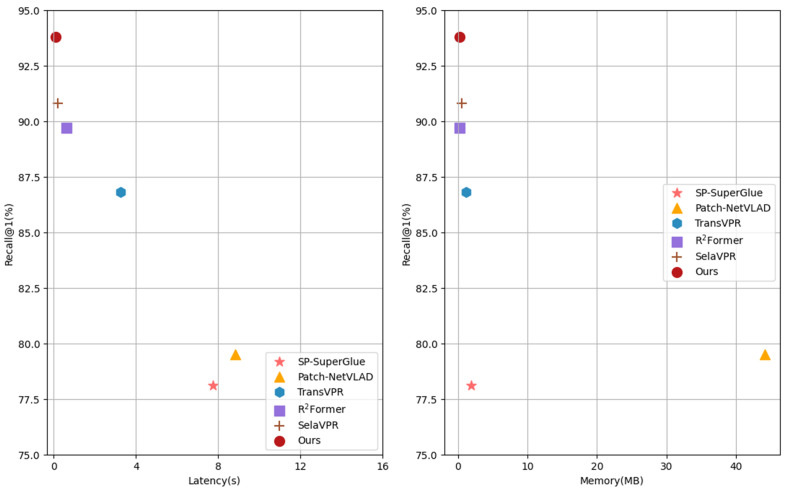
The efficiency–accuracy trade-off graphs for various methods on the MSLS val dataset.

Furthermore, Figure 4 depicts the efficiency–accuracy trade-off graphs for all two-stage methods. The vertical axis represents the R@1 values on the MSLS val dataset, while the horizontal axis represents the total time and memory consumption for processing a single query image, respectively. The total time consumption here refers to the sum of the feature extraction time and the matching latency for processing a single query image. It can be concluded from the data in the figure that our method performs the best, achieving the optimal trade-off between accuracy and efficiency in terms of time consumption and memory consumption. Our method provides an effective solution for the practical deployment of the VPR system in real-world scenarios.

#### 4.2.3. Loop Closure Detection Performance

In order to verify that our method can be applied to the practical loop closure detection tasks of robots, we selected two commonly used datasets for loop closure detection, New College [68] and City Centre [69], for experimental testing and compared ours with several state-of-the-art two-stage methods. In terms of evaluation metrics, we adopted the precision and Recall commonly used in loop closure detection to evaluate the performance of each method. In terms of the experimental setup, we selected the top-10 retrieval results for each query to calculate the maximum Recall at 100% precision and average time consumption. The experimental results are shown in Table 4. As demonstrated in the table, compared with SP-SuperGlue and SelaVPR, our method achieves significant absolute improvements in Recall values: a 12.9% and 3.4% increase, respectively, on the New College dataset, and a 15.9% and 5.5% gain on the City Centre dataset. In terms of time consumption, our method has achieved significant advantages. Compared to Patch-NetVLAD and SelaVPR, our method achieves speedups of 163.0 times and 2.6 times on the New College dataset, and 185.8 times and 2.9 times on the City Centre dataset, respectively. Overall, our method exhibits superior accuracy and efficiency in the loop closure detection tasks compared to other two-stage methods.

#### 4.2.4. Ablation Studies

Inspired by [70,71], to verify the effectiveness of each component of our proposed method, we conducted ablation experiments on the MSLS val dataset. The experimental results are shown in Table 5, where MM-MB represents the MobileMamba-Mona Block. From the first and second rows of the table, it can be observed that R@1 increased by an absolute 9.2% after incorporating the depth information, indicating that multimodal information is more robust than single RGB information in handling VPR problems. R@1 increased by 14.9% after the introduction of the MobileMamba-Mona Block, indicating that the Unified feature extractor can extract richer and more robust RGB and depth information compared to the original MobileMamba. According to the data in the table, with the introduction of the DWTF module, RGB information and depth information can be effectively integrated to generate more discriminative and robust multimodal descriptors, and R@1 has increased by an absolute 5.9%. It can be observed from the last two rows of data in the table that through the design of the SNGM module, effectively utilizing information such as geometric structure and spatial distance in the depth map to assist in the reranking can enhance the accuracy of the VPR task. Overall, through the effective extraction and integration of RGB and depth information as well as the design of the SNGM module, our method achieved the best performance, demonstrating the effectiveness of the design of each component of the proposed method.

## 5. Conclusions

We propose a novel coarse-to-fine VPR hierarchical framework that differs from conventional methods, which rely solely on RGB single-modal information for coarse retrieval and reranking. Our approach integrates depth information into the designed VPR model. The proposed Discrete Wavelet Transform Fusion (DWTF) module is utilized to achieve the deep integration of RGB information and depth information, forming a robust global multimodal descriptor for coarse retrieval. Meanwhile, the precise matching of depth features is accomplished by designing the Spiking Neuron Graph Matching (SNGM) reranking module, thereby enhancing the accuracy of VPR. Extensive experiments conducted on several benchmark datasets validate that the proposed method achieves state-of-the-art performance with lower computational time and memory consumption, providing a practical VPR solution that balances accuracy and efficiency. For practical deployment, our method supports flexible sensor configurations: it can operate with a full sensor suite (utilizing raw RGB-D/LiDAR data) or a minimal setup (relying solely on monocular cameras with estimated depth).

While our method demonstrates strong performance, some limitations remain. First, the dependence on monocular depth estimation may affect performance in textureless scenes. Second, extreme viewpoint changes (>40°) still pose challenges. Future work will explore hybrid depth sensing and viewpoint-adaptive feature learning to address these limitations.

## Figures and Tables

**Table 1:** Summary of the datasets. “+” indicates the presence of such changes in the dataset, while “−” indicates their absence.

Dataset	Environment			Variation					Number	
	Urban	Suburban	Natural	Viewpoint	Day/Night	Weather	Seasonal	Dynamic	Database	Queries
MSLS val [62]	✔	✔	✔	+	+	+	+	+	19k	740
MSLS Challenge [62]	✔	✔	✔	+	+	+	+	+	39k	27,092
Pitts30k [7]	✔			+	−	−	−	+	10k	6816
Tokyo 24/7 [63]	✔			+	+	−	−	+	76k	315

**Table 2:** Performance comparison with state-of-the-art methods on VPR benchmark datasets. The best results are highlighted in **bold**, while the second best ones are indicated with underlines.

Method	MSLS val			MSLS Challenge			Pitts30k Test			Tokyo 24/7		
	R@1	R@5	R@10	R@1	R@5	R@10	R@1	R@5	R@10	R@1	R@5	R@10
NetVLAD [7]	53.1	66.5	71.1	35.1	47.4	51.7	81.9	91.2	93.7	64.4	78.4	81.6
SFRS [9]	69.2	80.3	83.1	41.5	52.0	56.3	89.4	94.7	95.9	85.4	91.1	93.3
GCL [32]	80.9	90.7	92.6	62.3	76.2	81.1	79.2	90.4	93.2	58.1	74.3	78.1
SelaVPR (global) [19]	87.7	95.8	96.6	69.6	86.9	90.1	90.2	**96.1**	**97.1**	81.9	94.9	**96.5**
Ours (global)	**90.2**	**96.2**	**96.8**	**73.2**	**87.3**	**90.4**	**92.7**	95.4	96.8	**87.5**	**95.3**	96.2
SP-SuperGlue [45,65]	78.1	81.9	84.3	50.6	56.9	58.3	87.2	94.8	96.4	88.2	90.2	90.2
Patch-NetVLAD [15]	79.5	86.2	87.7	48.1	57.6	60.5	88.7	94.5	95.9	86.0	88.6	90.5
TransVPR [16]	86.8	91.2	92.4	63.9	74.0	77.5	89.0	94.9	96.2	-	-	-
$R^{2}\mathrm{Former}$ [18]	89.7	95.0	96.2	73.0	85.9	88.8	91.1	95.2	96.3	88.6	91.4	91.7
SelaVPR [19]	90.8	96.4	97.2	73.5	87.5	90.6	92.8	96.8	97.7	94.0	96.8	97.5
Ours	**93.8**	**97.3**	**97.5**	**80.9**	**89.3**	**91.3**	**94.3**	**97.2**	**98.1**	**97.8**	**98.3**	**99.1**

**Table 3:** The comparison of all two-stage methods in terms of feature extraction time, feature matching time, and memory usage. All methods are measured on the MSLS val using a single NVIDIA GeForce RTX 3090. The best results are highlighted in **bold**.

Method	Extraction Latency (ms)	Matching Latency (s)	Memory (MB)
SP-SuperGlue [45,65]	163	7.6	1.93
Patch-NetVLAD [15]	1330	7.5	44.14
TransVPR [16]	45	3.2	1.17
$R^{2}\mathrm{Former}$ [18]	27.4	0.59	**0.27**
SelaVPR [19]	36.2	0.15	0.52
Ours	**19.3**	**0.09**	0.29

**Table 4:** Comparison of loop closure detection performance of each two-stage method on the New College and City Centre datasets. The best results are highlighted in **bold**.

Method	New College		City Centre	
	Recall (%)	Time (ms)	Recall (%)	Time (ms)
SP-SuperGlue [45,65]	85.2	2367.2	79.1	2354.8
Patch-NetVLAD [15]	93.4	2819.4	86.3	2823.9
$R^{2}\mathrm{Former}$ [18]	91.5	193.5	85.4	186.4
SelaVPR [19]	94.7	45.6	89.5	44.5
Ours	**98.1**	**17.3**	**95.0**	**15.2**

**Table 5:** Ablation experiments on each component of the proposed method on the MSLS val dataset. The best results are highlighted in **bold**.

RGB	Depth	MM-MB	DWTF	SNGM	MSLS val		
					R@1	R@5	R@10
✔	×	×	×	×	64.2	69.4	75.1
✔	✔	×	×	×	73.4	77.7	79.5
✔	✔	✔	×	×	84.3	87.5	89.9
✔	✔	✔	✔	×	90.2	96.2	96.8
✔	✔	✔	✔	✔	**93.8**	**97.3**	**97.5**

## Data Availability

Data are contained within the article. Readers can refer to the following website for the datasets: (https://github.com/gmberton/VPR-datasets-downloader (accessed on 28 June 2025).

## References

[1] Lowry S., Sünderhauf N., Newman P., Leonard J.J., Cox D., Corke P., Milford M.J. (2015). Visual place recognition: A survey. IEEE Trans. Robot..

[2] Zaffar M., Garg S., Milford M., Kooij J., Flynn D., McDonald-Maier K., Ehsan S. (2021). Vpr-bench: An open-source visual place recognition evaluation framework with quantifiable viewpoint and appearance change. Int. J. Comput. Vis..

[3] Zhang X., Wang L., Su Y. (2021). Visual place recognition: A survey from deep learning perspective. Pattern Recognit..

[4] Masone C., Caputo B. (2021). A survey on deep visual place recognition. IEEE Access.

[5] Schubert S., Neubert P. (2021). What makes visual place recognition easy or hard?. arXiv.

[6] Garg S., Fischer T., Milford M. (2021). Where is your place, visual place recognition?. arXiv.

[7] Arandjelovic R., Gronat P., Torii A., Pajdla T., Sivic J. NetVLAD: CNN architecture for weakly supervised place recognition. Proceedings of the IEEE Conference on Computer Vision and Pattern Recognition.

[8] Jin Kim H., Dunn E., Frahm J.M. Learned contextual feature reweighting for image geo-localization. Proceedings of the IEEE Conference on Computer Vision and Pattern Recognition.

[9] Ge Y., Wang H., Zhu F., Zhao R., Li H. (2020). Self-supervising fine-grained region similarities for large-scale image localization. Proceedings of the Computer Vision–ECCV 2020: 16th European Conference.

[10] Peng G., Zhang J., Li H., Wang D. Attentional pyramid pooling of salient visual residuals for place recognition. Proceedings of the IEEE/CVF International Conference on Computer Vision.

[11] Peng G., Yue Y., Zhang J., Wu Z., Tang X., Wang D. Semantic reinforced attention learning for visual place recognition. Proceedings of the 2021 IEEE International Conference on Robotics and Automation (ICRA).

[12] Ali-Bey A., Chaib-Draa B., Giguere P. Mixvpr: Feature mixing for visual place recognition. Proceedings of the IEEE/CVF Winter Conference on Applications of Computer Vision.

[13] Ali-bey A., Chaib-draa B., Giguère P. (2022). Gsv-cities: Toward appropriate supervised visual place recognition. Neurocomputing.

[14] Ali-Bey A., Chaib-draa B., Giguère P. BoQ: A place is worth a bag of learnable queries. Proceedings of the IEEE/CVF Conference on Computer Vision and Pattern Recognition.

[15] Hausler S., Garg S., Xu M., Milford M., Fischer T. Patch-netvlad: Multi-scale fusion of locally-global descriptors for place recognition. Proceedings of the IEEE/CVF Conference on Computer Vision and Pattern Recognition.

[16] Wang R., Shen Y., Zuo W., Zhou S., Zheng N. TransVPR: Transformer-based place recognition with multi-level attention aggregation. Proceedings of the IEEE/CVF Conference on Computer Vision and Pattern Recognition.

[17] Fischler M.A., Bolles R.C. (1981). Random sample consensus: A paradigm for model fitting with applications to image analysis and automated cartography. Commun. ACM.

[18] Zhu S., Yang L., Chen C., Shah M., Shen X., Wang H. R2former: Unified retrieval and reranking transformer for place recognition. Proceedings of the IEEE/CVF Conference on Computer Vision and Pattern Recognition.

[19] Lu F., Zhang L., Lan X., Dong S., Wang Y., Yuan C. Towards Seamless Adaptation of Pre-trained Models for Visual Place Recognition. Proceedings of the Twelfth International Conference on Learning Representations.

[20] Lu F., Dong S., Zhang L., Liu B., Lan X., Jiang D., Yuan C. Deep homography estimation for visual place recognition. Proceedings of the AAAI Conference on Artificial Intelligence.

[21] Dharmasiri T., Spek A., Drummond T. (2018). Eng: End-to-end neural geometry for robust depth and pose estimation using cnns. Proceedings of the Asian Conference on Computer Vision.

[22] Sun L.C., Bhatt N.P., Liu J.C., Fan Z., Wang Z., Humphreys T.E., Topcu U. (2024). Mm3dgs slam: Multi-modal 3d gaussian splatting for slam using vision, depth, and inertial measurements. Proceedings of the 2024 IEEE/RSJ International Conference on Intelligent Robots and Systems (IROS).

[23] Yuan J., Zhu S., Tang K., Sun Q. (2022). ORB-TEDM: An RGB-D SLAM approach fusing ORB triangulation estimates and depth measurements. IEEE Trans. Instrum. Meas..

[24] Piasco N., Sidibé D., Gouet-Brunet V., Demonceaux C. (2019). Learning scene geometry for visual localization in challenging conditions. Proceedings of the 2019 International Conference on Robotics and Automation (ICRA).

[25] Piasco N., Sidibé D., Gouet-Brunet V., Demonceaux C. (2021). Improving image description with auxiliary modality for visual localization in challenging conditions. Int. J. Comput. Vis..

[26] Garg S., Babu M., Dharmasiri T., Hausler S., Suenderhauf N., Kumar S., Drummond T., Milford M. (2019). Look no deeper: Recognizing places from opposing viewpoints under varying scene appearance using single-view depth estimation. Proceedings of the 2019 International Conference on Robotics and Automation (ICRA).

[27] Hu H., Qiao Z., Cheng M., Liu Z., Wang H. (2020). Dasgil: Domain adaptation for semantic and geometric-aware image-based localization. IEEE Trans. Image Process..

[28] Khaliq A., Xu M., Hausler S., Milford M., Garg S. (2024). VLAD-BuFF: Burst-aware fast feature aggregation for visual place recognition. Proceedings of the European Conference on Computer Vision.

[29] Berton G., Masone C., Caputo B. Rethinking visual geo-localization for large-scale applications. Proceedings of the IEEE/CVF Conference on Computer Vision and Pattern Recognition.

[30] Ali-Bey A., Chaib-draa B., Giguère P. (2023). Global proxy-based hard mining for visual place recognition. arXiv.

[31] Berton G., Trivigno G., Caputo B., Masone C. Eigenplaces: Training viewpoint robust models for visual place recognition. Proceedings of the IEEE/CVF International Conference on Computer Vision.

[32] Leyva-Vallina M., Strisciuglio N., Petkov N. Data-efficient large scale place recognition with graded similarity supervision. Proceedings of the IEEE/CVF Conference on Computer Vision and Pattern Recognition.

[33] Izquierdo S., Civera J. Optimal transport aggregation for visual place recognition. Proceedings of the IEEE/CVF Conference on Computer Vision and Pattern Recognition.

[34] Lu F., Lan X., Zhang L., Jiang D., Wang Y., Yuan C. Cricavpr: Cross-image correlation-aware representation learning for visual place recognition. Proceedings of the IEEE/CVF Conference on Computer Vision and Pattern Recognition.

[35] Cao B., Araujo A., Sim J. (2020). Unifying deep local and global features for image search. Proceedings of the Computer Vision–ECCV 2020: 16th European Conference.

[36] Berton G., Masone C., Paolicelli V., Caputo B. Viewpoint invariant dense matching for visual geolocalization. Proceedings of the IEEE/CVF International Conference on Computer Vision.

[37] Hausler S., Moghadam P. (2025). Pair-vpr: Place-aware pre-training and contrastive pair classification for visual place recognition with vision transformers. IEEE Robot. Autom. Lett..

[38] Defferrard M., Bresson X., Vandergheynst P. (2016). Convolutional neural networks on graphs with fast localized spectral filtering. Adv. Neural Inf. Process. Syst..

[39] Wang R., Yan J., Yang X. Learning combinatorial embedding networks for deep graph matching. Proceedings of the IEEE/CVF International Conference on Computer Vision.

[40] Yu T., Wang R., Yan J., Li B. Learning deep graph matching with channel-independent embedding and hungarian attention. Proceedings of the International Conference on Learning Representations 2019.

[41] Babai L. (2018). Group, graphs, algorithms: The graph isomorphism problem. Proceedings of the International Congress of Mathematicians: Rio de Janeiro 2018.

[42] Yan J., Cho M., Zha H., Yang X., Chu S.M. (2015). Multi-graph matching via affinity optimization with graduated consistency regularization. IEEE Trans. Pattern Anal. Mach. Intell..

[43] Yan J., Wang J., Zha H., Yang X., Chu S. (2015). Consistency-driven alternating optimization for multigraph matching: A unified approach. IEEE Trans. Image Process..

[44] He J., Huang Z., Wang N., Zhang Z. (2024). Learnable Graph Matching: A Practical Paradigm for Data Association. IEEE Trans. Pattern Anal. Mach. Intell..

[45] Sarlin P.E., DeTone D., Malisiewicz T., Rabinovich A. Superglue: Learning feature matching with graph neural networks. Proceedings of the IEEE/CVF Conference on Computer Vision and Pattern Recognition.

[46] Mena G., Belanger D., Linderman S., Snoek J. (2018). Learning latent permutations with gumbel-sinkhorn networks. arXiv.

[47] Lin Y., Yang M., Yu J., Hu P., Zhang C., Peng X. Graph matching with bi-level noisy correspondence. Proceedings of the IEEE/CVF International Conference on Computer Vision.

[48] Wang R., Guo Z., Jiang S., Yang X., Yan J. Deep learning of partial graph matching via differentiable top-k. Proceedings of the IEEE/CVF Conference on Computer Vision and Pattern Recognition.

[49] Gao P., Zhang H. (2020). Long-term place recognition through worst-case graph matching to integrate landmark appearances and spatial relationships. Proceedings of the 2020 IEEE International Conference on Robotics and Automation (ICRA).

[50] Godard C., Mac Aodha O., Firman M., Brostow G.J. Digging into self-supervised monocular depth estimation. Proceedings of the IEEE/CVF International Conference on Computer Vision.

[51] Zhang N., Nex F., Vosselman G., Kerle N. Lite-mono: A lightweight cnn and transformer architecture for self-supervised monocular depth estimation. Proceedings of the IEEE/CVF Conference on Computer Vision and Pattern Recognition.

[52] Geiger A., Lenz P., Stiller C., Urtasun R. (2013). Vision meets robotics: The kitti dataset. Int. J. Robot. Res..

[53] He H., Zhang J., Cai Y., Chen H., Hu X., Gan Z., Wang Y., Wang C., Wu Y., Xie L. (2024). Mobilemamba: Lightweight multi-receptive visual mamba network. arXiv.

[54] Yin D., Hu L., Li B., Zhang Y., Yang X. (2024). 5% > 100%: Breaking performance shackles of full fine-tuning on visual recognition tasks. arXiv.

[55] Duan Y., Liu F., Jiao L., Zhao P., Zhang L. (2017). SAR image segmentation based on convolutional-wavelet neural network and Markov random field. Pattern Recognit..

[56] Li Z., Kuang Z.S., Zhu Z.L., Wang H.P., Shao X.L. (2022). Wavelet-based texture reformation network for image super-resolution. IEEE Trans. Image Process..

[57] Pu T., Ni G. (2000). Contrast-based image fusion using the discrete wavelet transform. Opt. Eng..

[58] Lai H., Yin P., Scherer S. (2022). Adafusion: Visual-lidar fusion with adaptive weights for place recognition. IEEE Robot. Autom. Lett..

[59] Zhang Y., Wu C., Zhang T., Zheng Y. (2024). Full-scale feature aggregation and grouping feature reconstruction based uav image target detection. IEEE Trans. Geosci. Remote Sens..

[60] Phung H., Dao Q., Tran A. Wavelet diffusion models are fast and scalable image generators. Proceedings of the IEEE/CVF Conference on Computer Vision and Pattern Recognition.

[61] Shi X., Hao Z., Yu Z. SpikingResformer: Bridging ResNet and vision transformer in spiking neural networks. Proceedings of the IEEE/CVF Conference on Computer Vision and Pattern Recognition.

[62] Warburg F., Hauberg S., Lopez-Antequera M., Gargallo P., Kuang Y., Civera J. Mapillary street-level sequences: A dataset for lifelong place recognition. Proceedings of the IEEE/CVF Conference on Computer Vision and Pattern Recognition.

[63] Torii A., Arandjelovic R., Sivic J., Okutomi M., Pajdla T. 24/7 place recognition by view synthesis. Proceedings of the IEEE Conference on Computer Vision and Pattern Recognition.

[64] Torii A., Sivic J., Pajdla T., Okutomi M. Visual place recognition with repetitive structures. Proceedings of the IEEE Conference on Computer Vision and Pattern Recognition.

[65] DeTone D., Malisiewicz T., Rabinovich A. Superpoint: Self-supervised interest point detection and description. Proceedings of the IEEE Conference on Computer Vision and Pattern Recognition Workshops.

[66] Zhong H., Chen Z., Qin C., Huang Z., Zheng V.W., Xu T., Chen E. (2020). Adam revisited: A weighted past gradients perspective. Front. Comput. Sci..

[67] Schroff F., Kalenichenko D., Philbin J. Facenet: A unified embedding for face recognition and clustering. Proceedings of the IEEE Conference on Computer Vision and Pattern Recognition.

[68] Smith M., Baldwin I., Churchill W., Paul R., Newman P. (2009). The new college vision and laser data set. Int. J. Robot. Res..

[69] Cummins M., Newman P. (2008). FAB-MAP: Probabilistic localization and mapping in the space of appearance. Int. J. Robot. Res..

[70] Ning J., Zhang Y., Zhao X., Coleman S., Li K., Kerr D. (2023). Samloc: Structure-aware constraints with multi-task distillation for long-term visual localization. Proceedings of the 2023 IEEE International Conference on Robotics and Automation (ICRA).

[71] Li K., Zhang Y., Ning J., Zhao X., Wang G., Liu W. (2024). Neighborhood Consensus Guided Matching Based Place Recognition with Spatial-Channel Embedding. Proceedings of the 2024 IEEE/RSJ International Conference on Intelligent Robots and Systems (IROS).

